# RSL3 Drives Ferroptosis through NF-*κ*B Pathway Activation and GPX4 Depletion in Glioblastoma

**DOI:** 10.1155/2021/2915019

**Published:** 2021-12-26

**Authors:** Shengbiao Li, Yuping He, Kexin Chen, Jiaojiao Sun, Lulu Zhang, Yaxin He, Hong Yu, Qiuhong Li

**Affiliations:** School of Basic Medical Sciences, Southwest Medical University, Luzhou 646000, China

## Abstract

Glioblastoma, the most aggressive form of malignant glioma, is very difficult to treat because of its aggressively invasive nature and high recurrence rates. RAS-selective lethal 3 (RSL3), a well-known inhibitor of glutathione peroxidase 4 (GPX4), could effectively induce oxidative cell death in glioblastoma cells through ferroptosis, and several signaling pathways are involved in this process. However, the role of the nuclear factor kappa-B (NF-*κ*B) pathway in glioblastoma cell ferroptosis has not yet been investigated. Therefore, we aimed to clarify the underlying mechanism of the NF-*κ*B pathway in RSL3-induced ferroptosis in glioblastoma cells. We found that RSL3 led to an increase in lipid ROS concentration and downregulation of ferroptosis-related proteins such as GPX4, ATF4, and SLC7A11 (xCT) in glioblastoma cells. Additionally, the NF-*κ*B pathway was activated by RSL3, and its inhibition by BAY 11-7082 could alleviate ferroptosis. The murine xenograft tumor model indicated that NF-*κ*B pathway inhibition could mitigate the antitumor effects of RSL3 *in vivo*. Furthermore, we found that GPX4 knockdown could not effectively induce ferroptosis. However, NF-*κ*B pathway activation coupled with GPX4 silencing induced ferroptosis. Additionally, ATF4 and xCT expression might be regulated by the NF-*κ*B pathway. Collectively, our results revealed that the NF-*κ*B pathway plays a novel role in RSL3-induced ferroptosis in glioblastoma cells and provides a new therapeutic strategy for glioblastoma treatment.

## 1. Introduction

Glioblastoma is the most aggressive form of malignant glioma with high recurrence rates and resistance to apoptosis [1]. Current therapies for glioblastoma include surgery, radiotherapy, and chemotherapy with temozolomide (TMZ) [2]. However, high-dose radiotherapy may lead to severe brain damage, and long-term chemotherapy treatment with TMZ is reported to result in drug resistance [3, 4]. Therefore, more innovative therapeutic strategies for glioblastoma are urgently needed.

Ferroptosis is a distinct form of regulated cell death that is largely dissimilar to other types of cell death, including apoptosis [5, 6]. It is closely related to several metabolic pathways involving iron, reactive oxygen species (ROS), and lipid metabolism [7, 8]. Most cancer cells exhibiting metabolic plasticity should be sensitive to ferroptosis inducers [9, 10]. Many ferroptosis inducers, such as GPX4- and system Xc^−^-inhibitors, are under clinical investigation across various tumor types [11]. The ferroptosis inducer, RSL3, triggers ferroptosis in various cancers by inhibiting GPX4 expression [12–14]. RSL3 can effectively induce ferroptosis in glioblastoma cells both *in vivo* and *in vitro* [13, 14]. However, the mechanism underlying RSL3-induced ferroptosis is not fully understood.

The NF-*κ*B pathway plays a pivotal role in the regulation of cell survival and proliferation [15, 16]. NF-*κ*B pathway inhibition combined with TMZ treatment results in more obvious apoptosis than TMZ alone in glioblastoma [17]. Additionally, the NF-*κ*B pathway is linked to ferroptosis: NF-*κ*B p65 phosphorylation suppresses ER stress-mediated ferroptosis, and p65 deletion contributes to intestinal epithelial cell ferroptosis [18], and NF-*κ*B signaling inhibition results in human breast cancer cell ferroptosis [19].

Initially, we discovered that the NF-*κ*B pathway was activated in RSL3-induced ferroptosis in glioblastoma cells, and it is fascinating to clarify its role in ferroptosis. RSL3-induced ferroptosis in glioblastoma cells was characterized by an increase in lipid ROS and a decrease in GPX4, ATF4, and xCT expression. Inhibition of the NF-*κ*B pathway by BAY 11-7082 mitigated RSL3-induced ferroptosis *in vitro*. Furthermore, we carried out a xenograft tumor assay and found that inhibition of the NF-*κ*B pathway also alleviated the antitumor effects of RSL3 *in vivo*. In addition, we observed that activation of the NF-*κ*B pathway combined with GPX4 depletion contributed to ferroptosis in glioblastoma cells. NF-*κ*B pathway activation was negatively related to the protein levels of ATF4 and xCT. This study reveals the vital role of the NF-*κ*B pathway in inducing ferroptosis in glioblastoma cells, and it might serve as a potential target for glioblastoma therapy.

## 2. Materials and Methods

### 2.1. Cell Culture and Reagents

The human glioblastoma cell lines, U87 and U251, were gifted by the Guangzhou Institutes of Biomedicine and Health (GIBH). Glioblastoma cell lines were cultured in Dulbecco's modified Eagle's medium (DMEM; Gibco, Carlsbad, CA, USA) supplemented with 1% streptomycin/puromycin (HyClone, Logan, UT, USA) and 10% fetal bovine serum (FBS; Gibco). The cells were maintained in a humidified environment at 37°C and 5% CO_2_. RSL3 and BAY 11-7082 were purchased from Selleck Chemicals (Houston, TX, USA).

### 2.2. Cell Viability Assay

Cell viability was evaluated using the Cell Counting Kit-8 (CCK8) assay (Bimake, Houston, TX, USA), according to the manufacturer's instructions. Glioblastoma cells were seeded in a 96-well plate at a density of 8 × 10^3^ cells/well, cultured for 24 h, and treated with the indicated compounds. The optical density (OD) value was measured at 450 nm using a microplate reader BioTek ELX800 (Gene Co., Ltd., Shanghai, China).

### 2.3. Small Interfering RNA (siRNA) Transfection

Confluent glioblastoma cells were seeded in a 24-well plate at 60%–80% confluence and transfected with 120 nM of the specific siRNA per well (RiboBio, Guangzhou, China) using the Lipofectamine® RNAiMAX reagent (Invitrogen; Carlsbad, CA, USA) according to the manufacturer's instructions. Cells were harvested after 72 h of siRNA treatment. The siRNA sequences used are listed in Supplementary Table 1.

### 2.4. Western Blot Analysis

Cells were washed, collected, and centrifuged at 300 × g at 4°C for 5 min and lysed with radioimmunoprecipitation lysis buffer (RIPA; Beyotime, Shanghai, China). Proteins were separated on 15% polyacrylamide gels and transferred to polyvinylidene fluoride membranes (PVDF; Millipore, Billerica, MA, USA). The membranes were blocked in 5% nonfat powdered milk (Beyotime, Shanghai, China) for 1 h at room temperature and incubated with primary antibodies at 4°C overnight. After washing the antibody adequately, the membranes were incubated with horseradish peroxidase- (HRP-) linked secondary antibodies for 1 h at room temperature. Proteins were visualized using a MINI CHEN™ 610 chemiluminescent imaging system (Beijing Sage Creation Science Co., Ltd., Beijing, China). The primary antibodies used in this study are listed in Supplementary Table 2.

### 2.5. Immunofluorescence

Cells were washed and fixed with 4% paraformaldehyde for 30 min at room temperature and permeabilized with 0.3% Triton X-100 for 15 min. Thereafter, cells were blocked in 10% FBS and incubated with a rabbit anti-p65 antibody (Cell Signaling Technology, Boston, MA, USA) at 4°C overnight. The cells were then washed and labeled with Alexa Fluor® 488 donkey anti-rabbit IgG (H+L) (Invitrogen) at a dilution of 1 : 2000 for 1 h at room temperature. Nuclei were stained with 4′,6-diamidino-2-phenylindole (DAPI; Sigma, St. Louis, MO, USA) for 5 min. Images were captured using a DMi8 fluorescence microscope (Leica Microsystems, Wetzlar, Germany).

### 2.6. Lipid Peroxidation Detection by Flow Cytometry

Cells were seeded in a 6-well plate at a density of 2.2 × 10^5^ cells/well and then treated with the corresponding compounds. Cells were then collected, washed twice with PBS, and incubated with 5 *μ*M BODIPY™ 581/591 C11 (Invitrogen) at 37°C for 30 min. Lipid peroxidation in glioblastoma cells was detected using a BD FACSVerse™ flow cytometer (BD Biosciences, Franklin Lakes, NJ, USA).

### 2.7. RT-qPCR

Total RNA was extracted using a TRIzol reagent (Solarbio, Shanghai, China) according to the manufacturer's instructions. One microgram of total RNA was reverse-transcribed into cDNA using a ReverTra Ace qPCR RT Kit (TOYOBO, Osaka, Japan), according to the manufacturer's instructions. RT-qPCR was performed with a SYBR® Premix Ex Taq™ Kit (Takara Bio, Kusatsu, Japan), according to the manufacturer's instructions, on a QuantStudio™ 5 Real-Time PCR system (Applied Biosystems, Carlsbad, CA, USA). Target gene expression was normalized to GAPDH and calculated using the 2^-*ΔΔ*Ct^ method. Primer sequences used are listed in Supplementary Table 3.

### 2.8. Murine Xenograft Tumor Model

Female B-NDG mice (4–6 weeks old, 16–20 g) were purchased from Biocytogen (Biocytogen Jiangsu Co., Ltd., Jiangsu, China) and housed under specific pathogen-free conditions. 5 × 10^6^ U87 cells were resuspended in 200 *μ*L PBS buffer and then inoculated into the left hind limb of each mouse. Once tumor volumes reached ≥50 mm^3^, the mice were randomly divided into four groups (*n* = 5): the control, RSL3-only, BAY-only, and RSL3 plus BAY groups. Chemicals were administered through intratumor injection (100 mg/kg for RSL3 and 1 mg/kg for BAY 11-7082) biweekly for two weeks. Tumor volumes were calculated using a Vernier caliper every 3 days using the following formula:

(1) $$\begin{array}{c} \text{Tumor volume}=\frac{\left(\text{length}\times {\text{width}}^{2}\right)}{2}. \end{array}$$

At the end of the experiment, all mice were anesthetized and sacrificed, and the tumors were harvested immediately. All procedures were performed according to the Animal Ethics Committee protocol approved for this study by Southwest Medical University.

### 2.9. Statistical Analyses

Experiments were performed thrice, and values are presented as the mean ± SD. Statistical differences were determined using unpaired two-tailed Student's *t-tests*. Statistical significance was set at *p* < 0.05.

## 3. Results

### 3.1. RSL3 Treatment Increased Lipid ROS and Decreased Key Ferroptosis-Related Protein Expression in Glioblastoma Cells

We first measured the sensitivity of glioblastoma cells to different drugs (Supplementary Figure 1). TMZ, one of the most commonly used clinical drugs for glioblastoma treatment, had poor inhibitory effects on U87 cell proliferation. However, RSL3, an inhibitor of GPX4, could severely hinder glioblastoma cell proliferation. Other ferroptosis-induced compounds such as Erastin or FIN56 could hardly induce apparent cell death. Therefore, we tried to explore the mechanism of RSL3-induced ferroptosis in U87 glioblastoma cells.

**Figure 1:**
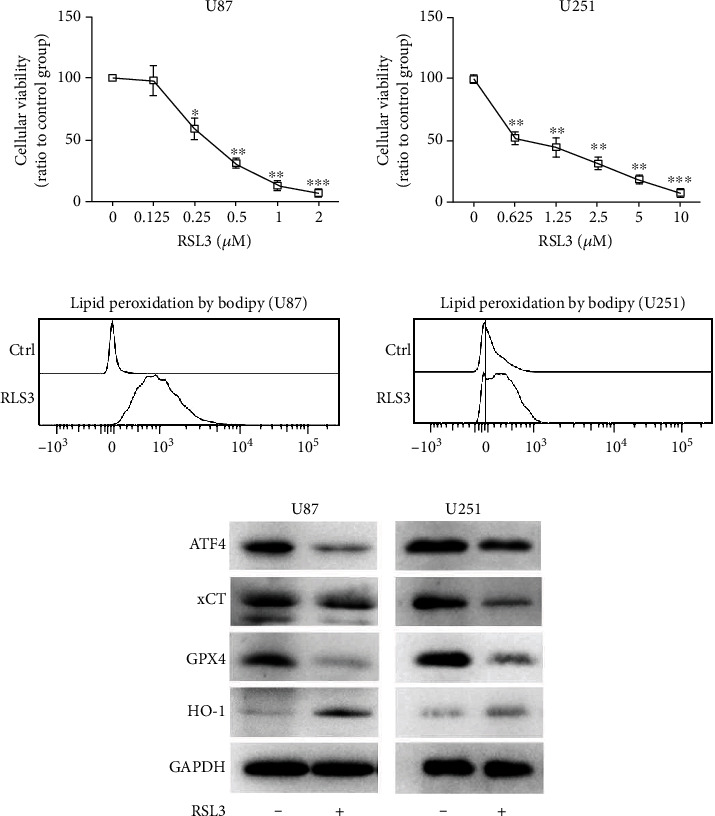
RSL3 upregulated lipid ROS and downregulated key ferroptosis protein expression in glioblastoma cells. (a) CCK-8 assay examining U87 and U251 cell viability at different concentrations of RSL3 treatment for 24 h. (b) Lipid peroxidation analysis for U87 and U251 treated with 0.25 *μ*M and 0.5 *μ*M RSL3 for 24 h, respectively. (c) Western blot analysis of ATF4, xCT, GPX4, and HO-1 expression in U87 and U251 treated with 0.25 *μ*M and 0.5 *μ*M RSL3 for 24 h, respectively. Experiments were repeated thrice, and data is presented as the mean ± SD. ^∗^*p* < 0.05,  ^∗∗^*p* < 0.01, and^∗∗∗^*p* < 0.001.

Two human glioblastoma cell lines, U87 and U251, were treated with RSL3 at the indicated concentrations for 24 h, respectively. We found that RSL3 induced cell death in a dose-dependent manner and U87 was more sensitive to RSL3 than U251 (Figure 1(a)). Additionally, lipid peroxidation, a typical feature of ferroptosis, was detected after RSL3 treatment (Figure 1(b)). Ferroptosis-related protein expression was examined by western blotting (Figure 1(c)). GPX4 expression was reduced after RSL3 treatment in both U87 and U251 cells, corresponding to results obtained by other studies [12]. Moreover, RSL3 treatment reduced ATF4 and xCT expression. HO-1 expression was upregulated in RSL3-treated cells; however, it is considered to play a dual role in ferroptosis promotion/inhibition [20]. However, the mRNA levels of ATF4, xCT, and HO-1 remained unchanged in U87 and U251 cells after RSL3 treatment (Supplementary Figure 2). Therefore, RSL3 treatment could effectively induce ferroptosis in glioblastoma cells.

### 3.2. NF-*κ*B Pathway Was Activated in RSL3-Treated Glioblastoma Cells

**Figure 2:**
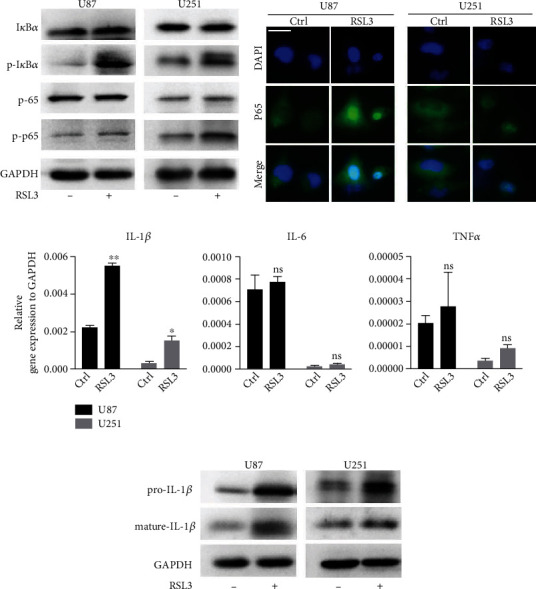
The NF-*κ*B pathway was activated in RSL3-induced ferroptosis. (a) The protein levels of NF-*κ*B pathway after RSL3 treatment. (b) Cellular localization of p65 was analyzed by immunofluorescence. Scale bar: 25 *μ*m. The mRNA and protein levels of IL-1*β* were determined by RT-qPCR (c) and western blotting (d). U87 and U251 cells were treated with 0.25 *μ*M and 0.5 *μ*M RSL3 for 24 h, respectively. Experiments were repeated thrice, and data is presented as the mean ± SD. ^∗^*p* < 0.05; ^∗∗^*p* < 0.01.

To examine whether the NF-*κ*B pathway is involved in RSL3-induced ferroptosis, the expression of NF-*κ*B pathway-related proteins was investigated. Phosphorylated p65 and I*κ*B*α* levels increased after RSL3 treatment (Figure 2(a)). Additionally, nuclear localization of p65 was observed in RSL3-treated glioblastoma cells by immunofluorescence (Figure 2(b)). Therefore, the NF-*κ*B pathway was activated in RSL3-induced ferroptosis.

Since NF-*κ*B pathway activation could be mediated by several inflammation-related factors, including IL-1*β*, IL-6, and TNF*α*, we further investigated the expression of these cytokines. *IL-1β* expression was significantly increased by RSL3 treatment, but *IL-6* or *TNFα* expression was not increased (Figure 2(c)). Moreover, pro-IL-1*β* and mature IL-1*β* protein levels simultaneously increased in glioblastoma cells (Figure 2(d)).

**Figure 3:**
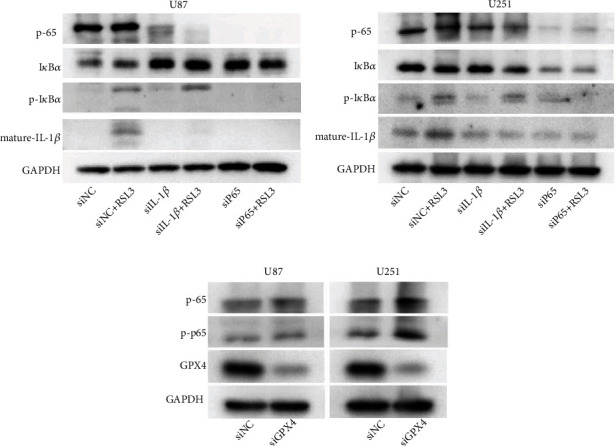
NF-*κ*B pathway activation was not mediated by IL-1*β* release or GPX4 depletion. (a, b) Western blot analysis of NF-*κ*B pathway-related proteins and mature IL-1*β* expression in U87 and U251 cells. (c) Total and phosphorylated p65 expression after GPX4 knockdown. U87 and U251 cells were treated with 0.25 *μ*M and 0.5 *μ*M RSL3 for 24 h, respectively. Experiments were repeated thrice.

Next, we silenced IL-1*β* with siRNA transfection to explore its regulatory role in the NF-*κ*B pathway (Figures 3(a) and 3(b)). IL-1*β* knockdown slightly increased phosphorylated I*κ*B*α* protein levels in RSL3-treated glioblastoma cells. However, p65 silencing did not upregulate mature IL-1*β* levels, indicating that increased IL-1*β* might not be related to NF-*κ*B pathway activation. We then silenced GPX4 by siRNA transfection to explore its influence on the NF-*κ*B pathway (Figure 3(c)). The intensity of phosphorylated p65 normalized to total p65 exhibited no significant difference between the siNC and siGPX4 groups (data not shown). Therefore, GPX4 depletion did not contribute to NF-*κ*B pathway activation.

### 3.3. NF-*κ*B Pathway Inhibition Reduced RSL3-Induced Ferroptosis in Glioblastoma Cells

**Figure 4:**
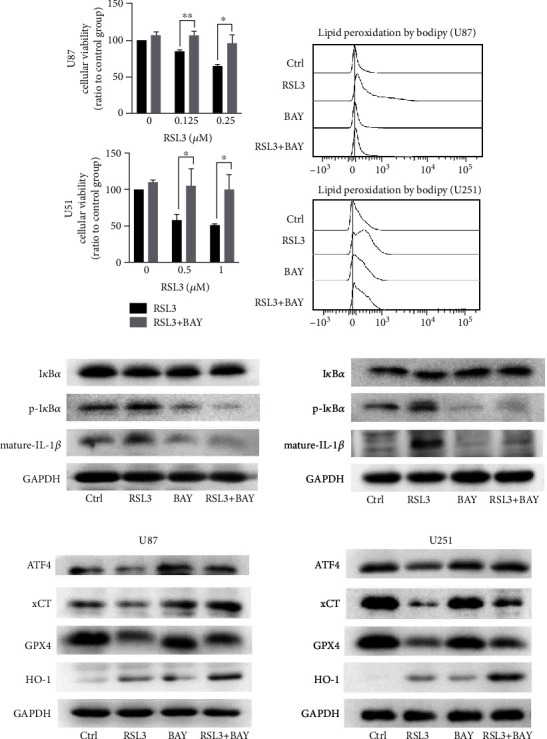
NF-*κ*B pathway inhibition, using BAY 11-7082 (12.5 *μ*M), reduced RSL3-induced ferroptosis in glioblastoma cells. (a) CCK-8 assay examined U87 and U251 cell viability at different concentrations of RSL3 for 24 h. (b) Lipid peroxidation analysis for U87 and U251 cells treated with 0.25 *μ*M and 0.5 *μ*M RSL3 for 24 h, respectively. (c) Western blot analysis of I*κ*B*α*, p-I*κ*B*α*, and mature IL-1*β* expression in U87 and U251 cells treated with 0.25 *μ*M and 0.5 *μ*M RSL3 for 24 h, respectively (d) Western blot analysis of ATF4, xCT, GPX4, and HO-1 expression in U87 and U251 cells treated with 0.25 *μ*M and 0.5 *μ*M RSL3 for 24 h, respectively. Experiments were performed thrice, and data is presented as the mean ± SD. ^∗^*p* < 0.05;  ^∗∗^*p* < 0.01.

To clarify whether the NF-*κ*B pathway activation is vital to RSL3-induced ferroptosis, we obstructed the NF-*κ*B pathway by its specific inhibitor BAY 11-7082 (BAY) and examined the effect on ferroptosis. RSL3-induced ferroptosis was inhibited by NF-*κ*B pathway inhibition (Figure 4(a)). In RSL3-treated glioblastoma cells, lipid ROS production was severely reduced by the addition of BAY-inhibitor (Figure 4(b)). Furthermore, phosphorylated I*κ*B*α* and mature IL-1*β* were expression markedly reduced (Figure 4(c)). However, ferroptosis-related protein expression, such as ATF4, xCT, and HO-1, increased (Figure 4(d) and Supplementary Figure 3). These results indicated that the inhibition of the NF-*κ*B pathway suppressed RSL3-induced ferroptosis in glioblastoma cells.

### 3.4. NF-*κ*B Pathway Inhibition Alleviated RSL3 Antitumor Effects in a Murine U87 Xenograft Model

**Figure 5:**
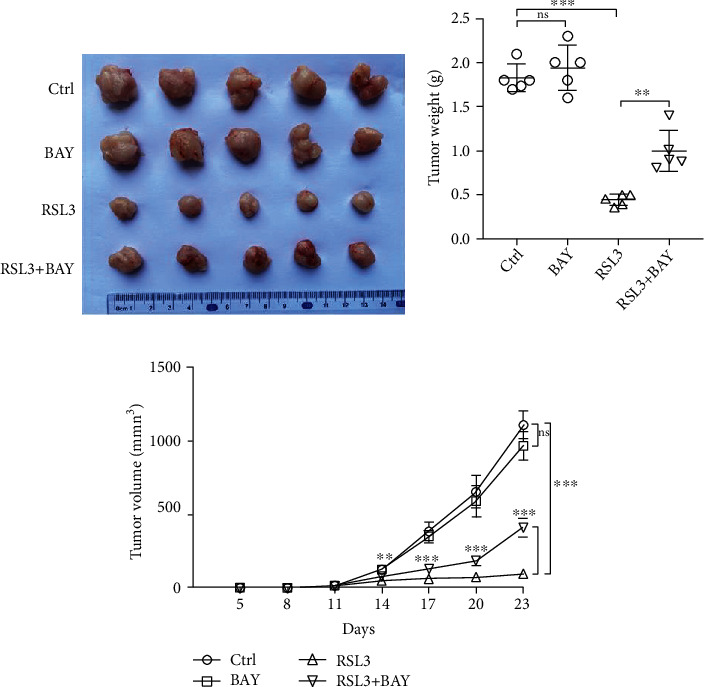
BAY 11-7082 (1 mg/kg) mitigates the antitumor effects of RSL3 (100 mg/kg) in a U87 xenograft model. (a) Representative images of tumors obtained from different groups at the termination of the xenograft assay. (b) Tumor weights from B-NDG mice injected with U87 cells and treated with different conditions. (c) Tumor volume growth curves of B-NDG mice in different treatment groups. Data are represented as the mean ± SD. ^∗∗^*p* < 0.01;  ^∗∗∗^*p* < 0.001.

We conducted a murine U87 xenograft assay to verify the results obtained *in vitro*. Tumor growth was strongly inhibited by RSL3 treatment, but NF-*κ*B pathway suppression by BAY 11-7082 partially alleviated RSL3 antitumor effects (Figure 5(a)). The weights of RSL3-treated tumors were approximately one-quarter of that in the control group, but cotreatment of RSL3 and BAY 11-7082 resulted in a significant increase in tumor weights (Figure 5(b)). The tumor growth curves correlated with the abovementioned results (Figure 5(c)).

### 3.5. GPX4 Silencing Did Not Trigger Ferroptosis in Glioblastoma Cells unless the NF-*κ*B Pathway Was Activated Simultaneously

**Figure 6:**
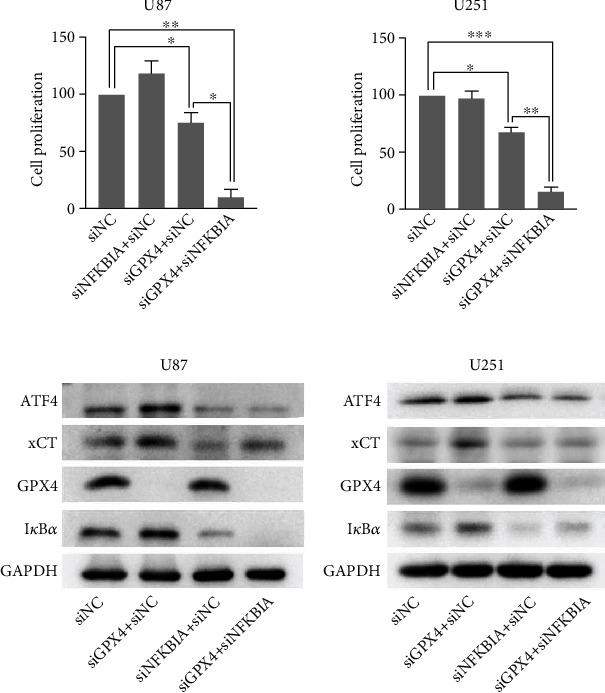
GPX4 silencing did not induce ferroptosis unless the NF-*κ*B pathway was activated simultaneously. (a) CCK-8 assay examining cell viability after siRNA (120 nM) treatment for 72 h. Data is presented as the mean ± SD. ^∗^*p* < 0.05,  ^∗∗^*p* < 0.01, and^∗∗∗^*p* < 0.001. (b) Western blot analysis of ferroptosis-related protein expression after siRNA treatment. Experiments were performed thrice.

RSL3 is known to induce ferroptosis by directly inhibiting GPX4 [12]. To verify whether GPX4 depletion alone would result in ferroptosis, we silenced GPX4 expression and examined the viability of glioblastoma cells. GPX4 silencing had a moderate effect on cell proliferation, but NF-*κ*B pathway activation by NFKBIA (IKB*α*) knockdown resulted in significant growth inhibition (Figure 6(a)).

**Figure 7:**
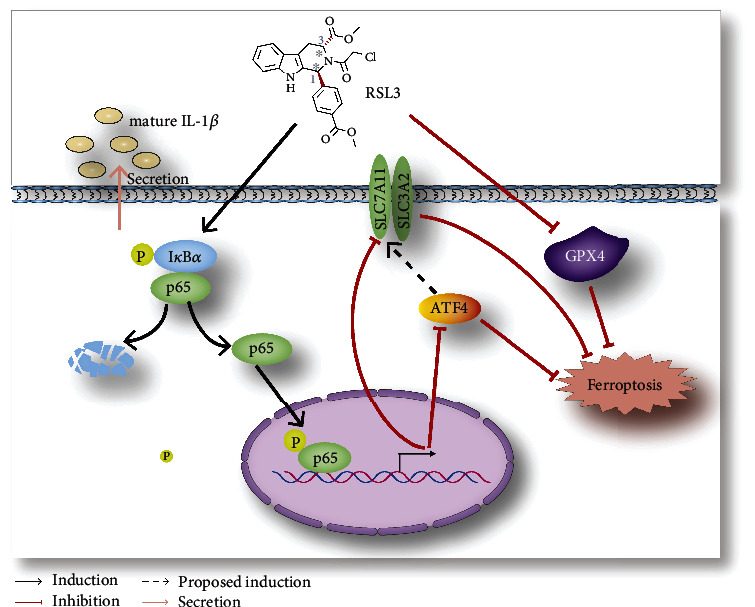
The possible mechanism of RSL3-induced ferroptosis in glioblastoma cells.

Next, we examined ferroptosis-related protein expression (Figure 6(b)). ATF4 and xCT protein levels remained the same in GPX4-depleted cells, but their expression was downregulated in RSL3-treated glioblastoma cells. Additionally, NF-*κ*B pathway activation decreased ATF4 and xCT expression in GPX4-depleted cells. Therefore, NF-*κ*B pathway activation was essential for glioblastoma cell ferroptosis and might mediate ATF4 and xCT expression (Figure 7).

## 4. Discussion

Unlike other types of regulated cell death, ferroptosis is mainly driven by lipid peroxidation [5, 6, 21]. GPX4 is an essential glutathione peroxidase that reduces lipid peroxidation [6, 12]. RSL3, an inhibitor of GPX4, has been reported to induce ferroptosis in various cancers such as glioblastoma [13, 14, 22]; however, the underlying mechanism is not yet fully understood.

Here, we aimed to investigate the underlying mechanisms of RSL3-induced ferroptosis in glioblastoma cells. We found that the NF-*κ*B pathway was activated in RSL3-treated glioblastoma cells, and NF-*κ*B pathway inhibition could prevent RSL3-induced ferroptosis; however, GPX4 silencing alone did not induce ferroptosis, but combining NF-*κ*B pathway activation with GPX4 depletion induced ferroptosis. Thus, we concluded that NF-*κ*B pathway activation is vital for inducing ferroptosis in glioblastoma cells.

NF-*κ*B pathway activation is critical for tumor survival [23]. When the NF-*κ*B pathway is obstructed, glioblastoma cells undergo apoptosis [16] and breast cancer cells experience ferroptosis [19]. In contrast, we observed that NF-*κ*B pathway activation contributes to RSL3-induced ferroptosis, and NF-*κ*B pathway inhibition by BAY 11-7082 prevents glioblastoma cell ferroptosis. Furthermore, we found that decreased ATF4 and xCT expression in RSL3-treated cells could be prevented by NF-*κ*B pathway inhibition, and NF-*κ*B pathway activation in GPX4-depleted glioblastoma cells reduced ATF4 and xCT expression. ATF4 activation has been reported to increase xCT expression in glioblastoma cells, and ATF4 silencing renders tumor cells susceptible to RSL3-induced ferroptosis [13]. Therefore, we concluded that the NF-*κ*B pathway facilitates ferroptosis by downregulating ATF4 and xCT in glioblastoma cells.

NF-*κ*B pathway activation results in the upregulation of inflammation-related factors, such as IL-1*β*, IL-6, and TNF*α*, which could further activate NF-*κ*B signaling [24, 25]. We examined the mRNA levels of IL-1*β*, IL-6, and TNF*α*. *IL-1β* expression increased after RSL3 treatment. NF-*κ*B pathway inhibition by BAY 11-7082 resulted in a decrease in mature IL-1*β*. IL-1*β* silencing, however, did not increase the phosphorylated I*κ*B*α* levels in RSL3-treated glioblastoma cells, implying that IL-1*β* expression is regulated by the NF-*κ*B pathway, the activation of which is independent of IL-1*β* (Figure 2(c)).

GPX4 overexpression disturbs NF-*κ*B pathway activation [26], but the effects of GPX4 depletion on the NF-*κ*B pathway remain unknown. We observe that GPX4 knockdown did not activate the NF-*κ*B pathway, suggesting that GPX4 inhibition does not activate the NF-*κ*B pathway in RSL3-induced ferroptosis. ROS [27] and the products of lipid peroxidation, such as 4-hydroxynonenal (4HNE) [28], could facilitate NF-*κ*B pathway activation; thus, we presumed that NF-*κ*B pathway activation might be attributed to the increased lipid ROS induced by RSL3.

GPX4 knockdown alone can trigger ferroptosis in human fibrosarcoma [12], but our results indicate that GPX4 depletion is not sufficient to induce ferroptosis in glioblastoma, unless the NF-*κ*B pathway is activated simultaneously. These contradictory results may be partly due to the different cancer types. Moreover, we found that increased ATF4 and xCT expression correlates with GPX4 knockdown, while ATF4 and xCT are considered to play a protective role against ferroptosis in glioblastoma cells [13]. Thus, we conclude that ATF4 and xCT upregulation disrupts ferroptosis induced by RNAi-mediated GPX4 knockdown, and NF-*κ*B pathway activation inhibits ATF4 and xCT expression and promotes ferroptosis.

## 5. Conclusions

NF-*κ*B pathway activation is vital for RSL3-induced ferroptosis in glioblastoma cells both *in vitro* and *in vivo*. Furthermore, RNAi-mediated GPX4 silencing cannot trigger ferroptosis in glioblastoma cells unless the NF-*κ*B pathway is activated simultaneously. Finally, NF-*κ*B pathway activation promotes ferroptosis by downregulating the expression of ATF4 and xCT. Therefore, we propose that the NF-*κ*B pathway might serve as a potential target when combined with radio- or chemotherapy in clinical treatment.

## Data Availability

All data included in this study are available from the corresponding authors upon request.

## Supplementary Materials

Supplementary Materials Supplementary Table 1: list of siRNA target sequences. Supplementary Table 2: list of antibodies used. Supplementary Table 3: list of RT-qPCR primers.

## References

[1] Hombach-Klonisch S., Mehrpour M., Shojaei S. (2018). Glioblastoma and chemoresistance to alkylating agents: involvement of apoptosis, autophagy, and unfolded protein response. *Pharmacology & Therapeutics*.

[2] Mittal S., Pradhan S., Srivastava T. (2015). Recent advances in targeted therapy for glioblastoma. *Expert Review of Neurotherapeutics*.

[3] Jiapaer S., Furuta T., Tanaka S., Kitabayashi T., Nakada M. (2018). Potential strategies overcoming the temozolomide resistance for glioblastoma. *Neurologia Medico-Chirurgica*.

[4] Huang C., Chen D., Zhu H., Lv S., Li Q., Li G. (2019). LITAF enhances radiosensitivity of human glioma cells via the FoxO1 pathway. *Cellular and Molecular Neurobiology*.

[5] Yang W. S., Stockwell B. R. (2016). Ferroptosis: death by lipid peroxidation. *Trends in Cell Biology*.

[6] Dixon S. J., Lemberg K. M., Lamprecht M. R. (2012). Ferroptosis: an iron-dependent form of nonapoptotic cell death. *Cell*.

[7] Li J., Cao F., Yin H. L. (2020). Ferroptosis: past, present and future. *Cell Death & Disease*.

[8] Tang D., Chen X., Kang R., Kroemer G. (2021). Ferroptosis: molecular mechanisms and health implications. *Cell Research*.

[9] Friedmann Angeli J. P., Krysko D. V., Conrad M. (2019). Ferroptosis at the crossroads of cancer-acquired drug resistance and immune evasion. *Nature Reviews. Cancer*.

[10] Shen Z., Song J., Yung B. C., Zhou Z., Wu A., Chen X. (2018). Emerging strategies of cancer therapy based on ferroptosis. *Advanced Materials*.

[11] Chen X., Kang R., Kroemer G., Tang D. (2021). Broadening horizons: the role of ferroptosis in cancer. *Nature Reviews. Clinical Oncology*.

[12] Yang W. S., SriRamaratnam R., Welsch M. E. (2014). Regulation of ferroptotic cancer cell death by GPX4. *Cell*.

[13] Chen D., Fan Z., Rauh M., Buchfelder M., Eyupoglu I. Y., Savaskan N. (2017). ATF4 promotes angiogenesis and neuronal cell death and confers ferroptosis in a xCT-dependent manner. *Oncogene*.

[14] Wang X., Lu S., He C. (2019). RSL3 induced autophagic death in glioma cells via causing glycolysis dysfunction. *Biochemical and Biophysical Research Communications*.

[15] Kim H. J., Hawke N., Baldwin A. S. (2006). NF-*κ*B and IKK as therapeutic targets in cancer. *Cell Death and Differentiation*.

[16] Zanotto-Filho A., Braganhol E., Schröder R. (2011). NF*κ*B inhibitors induce cell death in glioblastomas. *Biochemical Pharmacology*.

[17] Avci N. G., Ebrahimzadeh-Pustchi S., Akay Y. M. (2020). NF-*κ*B inhibitor with Temozolomide results in significant apoptosis in glioblastoma via the NF-*κ*B(p65) and actin cytoskeleton regulatory pathways. *Scientific Reports*.

[18] Xu M., Tao J., Yang Y. (2020). Ferroptosis involves in intestinal epithelial cell death in ulcerative colitis. *Cell Death & Disease*.

[19] Chang L. C., Chiang S. K., Chen S. E., Yu Y. L., Chou R. H., Chang W. C. (2018). Heme oxygenase-1 mediates BAY 11-7085 induced ferroptosis. *Cancer Letters*.

[20] Chiang S. K., Chen S. E., Chang L. C. (2019). A dual role of heme oxygenase-1 in cancer cells. *International journal of molecular sciences*.

[21] Dixon S. J., Stockwell B. R. (2014). The role of iron and reactive oxygen species in cell death. *Nature Chemical Biology*.

[22] Bush N. A., Chang S. M., Berger M. S. (2017). Current and future strategies for treatment of glioma. *Neurosurgical Review*.

[23] Zhang Q., Lenardo M. J., Baltimore D. (2017). 30 Years of NF-*κ*B: A Blossoming of Relevance to Human Pathobiology. *Cell*.

[24] Baeuerle P. A., Baltimore D. (1996). NF-*κ*B: Ten Years After. *Cell*.

[25] Barnes P. J., Karin M. (1997). Nuclear Factor-*κ*B — a pivotal transcription factor in chronic inflammatory diseases. *The New England Journal of Medicine*.

[26] Brigelius-Flohé R., Friedrichs B., Maurer S., Schultz M., Streicher R. (1997). Interleukin-1-induced nuclear factor *κ*B activation is inhibited by overexpression of phospholipid hydroperoxide glutathione peroxidase in a human endothelial cell line. *The Biochemical Journal*.

[27] Morgan M. J., Liu Z. G. (2011). Crosstalk of reactive oxygen species and NF-*κ*B signaling. *Cell Research*.

[28] Jang E. J., Kim D. H., Lee B. (2016). Activation of proinflammatory signaling by 4-hydroxynonenal-Src adducts in aged kidneys. *Oncotarget*.

