# Supplementary material for: RSL3 Drives Ferroptosis through NF-κB Pathway Activation and GPX4 Depletion in Glioblastoma
Source: Oxid Med Cell Longev. 2021 Dec 26;2021:2915019. doi: 10.1155/2021/2915019 (PMC8720588; doi:10.1155/2021/2915019)
Supplement: Supplementary Materials — Supplementary Table 1: list of siRNA target sequences. Supplementary Table 2: list of antibodies used. Supplementary Table 3: list of RT-qPCR primers. [file 2915019.f1.doc]

**Supplementary Figure 1**


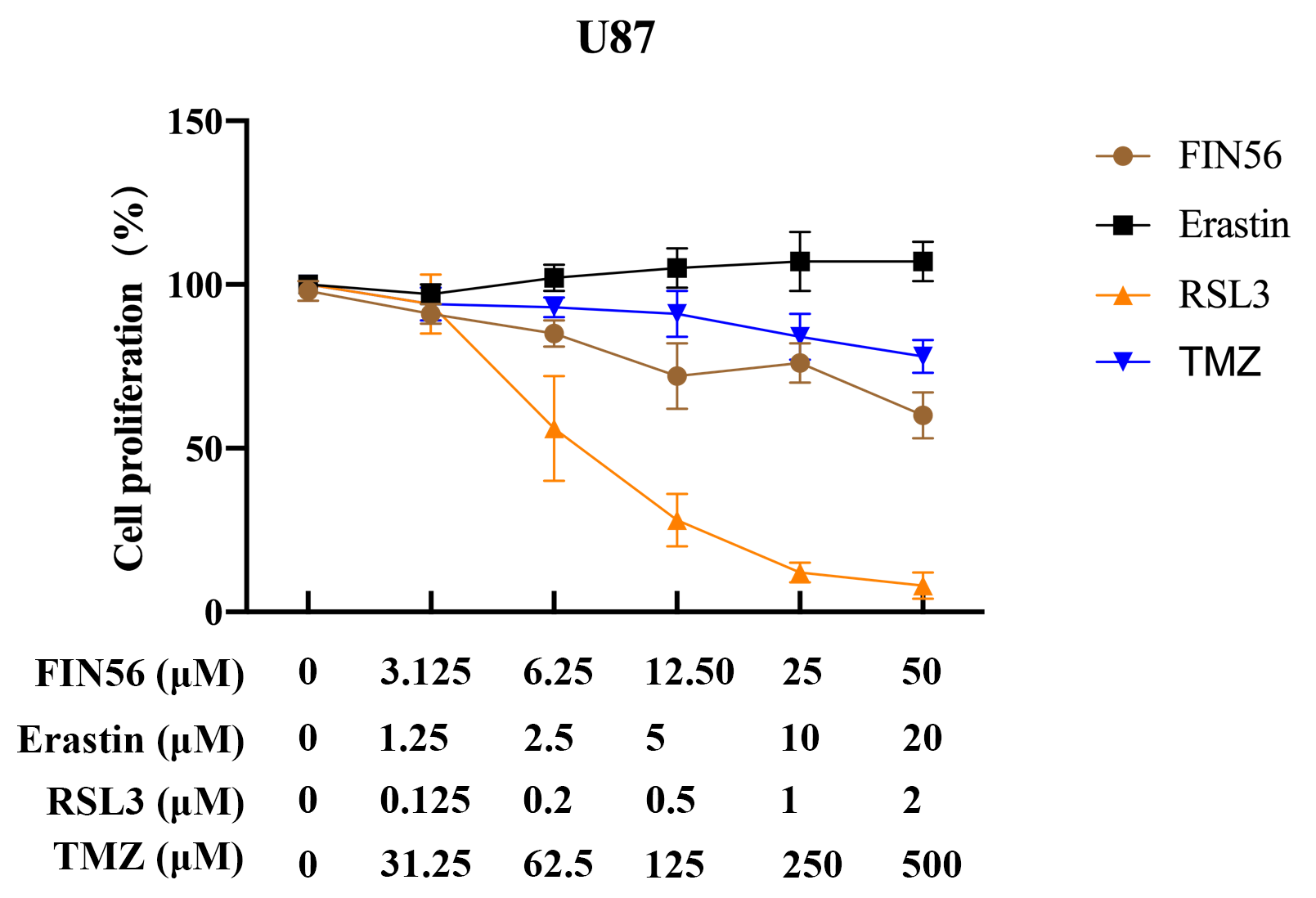


Supplementary Figure 1: Cellular viability of U87 glioblastoma cells treated with different compounds at 24 hours was measured by CCK-8 assay. Data are presented as mean ± SD.

**Supplementary Figure 2**


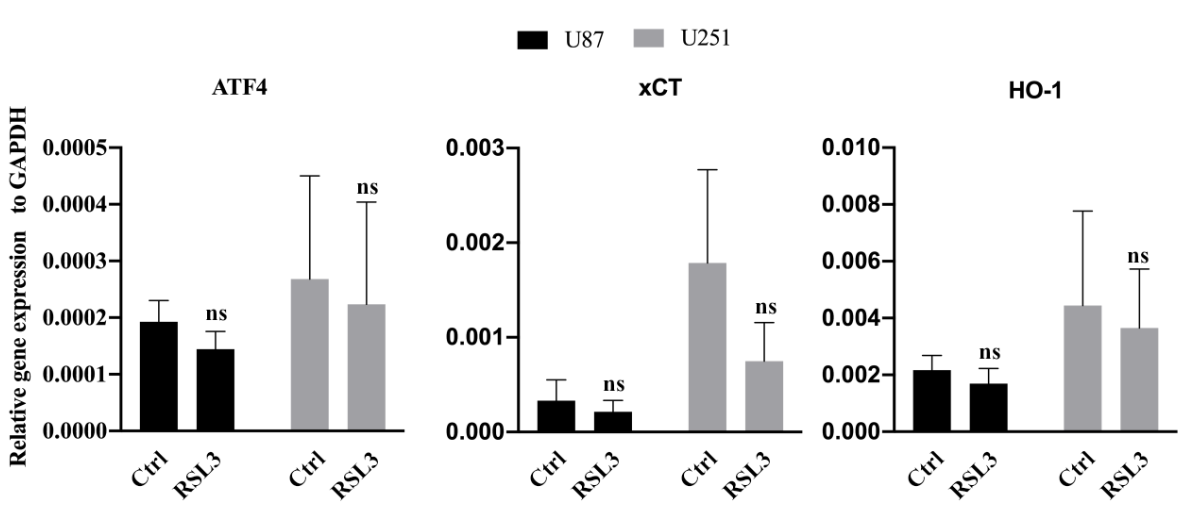


Supplementary Figure 2: qRT-PCR analysis of ATF4, xCT and HO-1 mRNA expression in U87 and U251 cells after treatment with 0.25 μM and 0.5 μM RSL3 for 24 h, respectively. Experiments were performed from three biological repeats. Data were presented as mean ± SD.

**Supplementary Figure 3**


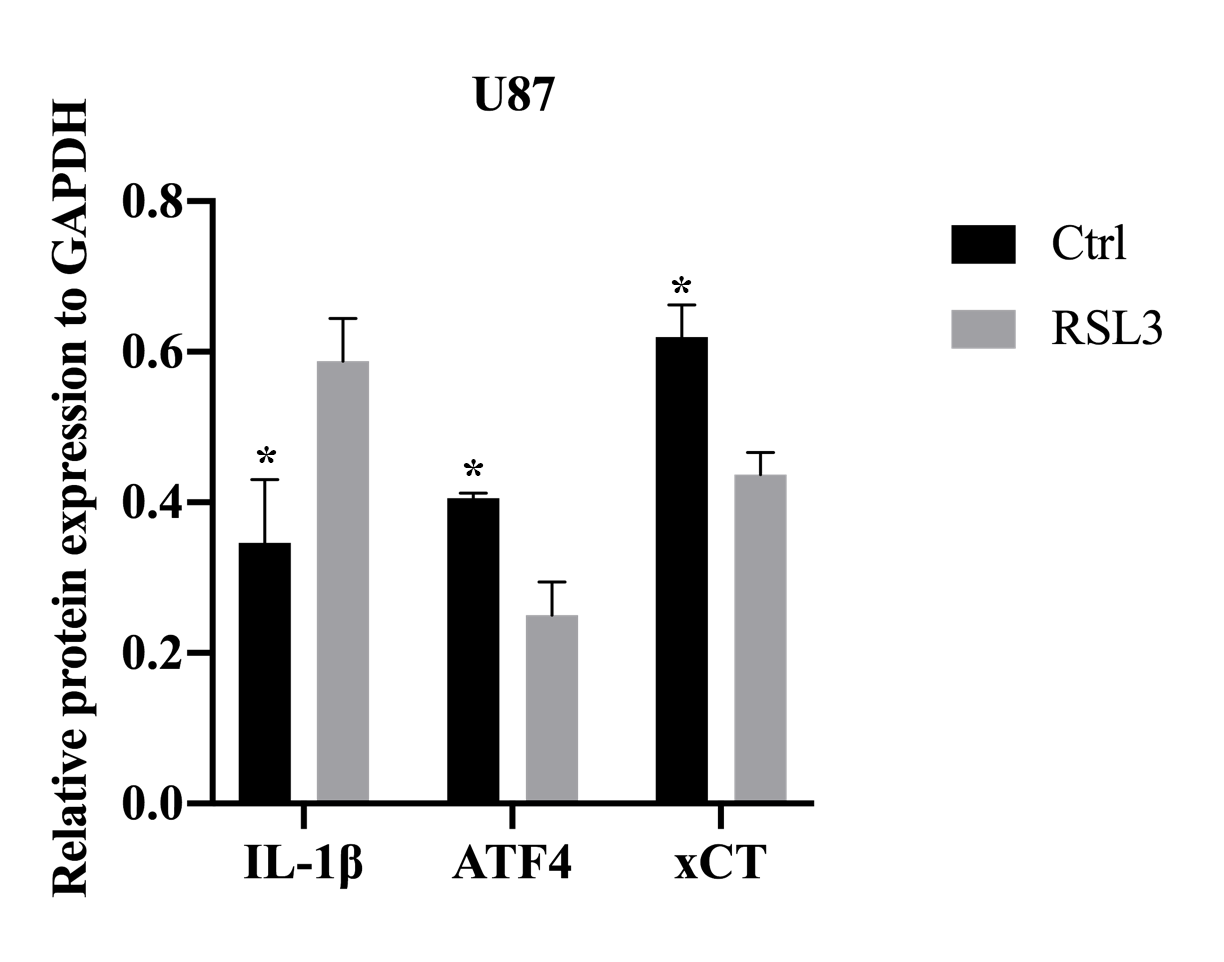


Supplementary Figure 3: Quantitative western blotting for IL-1β, ATF4 and xCT in RSL3-treated U87 for 24 h.

**Supplementary Table 1: list of siRNA target sequences.**

| Name of siRNA | Target sequence |
| --- | --- |
| siGPX4-1  siGPX4-2  siGPX4-3 | GCTACAACGTCAAATTCGA  GTAACGAAGAGATCAAAGA  GAGGCAAGACCGAAGTAAA |
| sip65-1  sip65-2  sip65-3 | GATTGAGGAGAAACGTAAA  CCCACGAGCTTGTAGGAAA  GCATCCAGACCAACAACAA |
| siNFKBIA-1  siNFKBIA-2  siNFKBIA-3 | CCACACGTGTCTACACTTA  CTCCGAGACTTTCGAGGAA  GCACTGACCATGGAAGTGA |
| siIL-1β-1  siIL-1β-2  siIL-1β-3 | CGATGCACCTGTACGATCA  GATGTCTGGTCCATATGAA  GGATGACTTGTTCTTTGAA |

**Supplementary Table 2: list of antibodies used.**

| Name of Antibody | Company（Cat. No.） | Dilution |
| --- | --- | --- |
| Mouse anti NF-κB p65 | Santa Cruz Biotechnology (sc-514451) | 1:1000 |
| Mouse anti p-NF-κB p65 (49.Ser 311) | Santa Cruz Biotechnology (sc-135769) | 1:1000 |
| Rabbit anti Heme Oxygenase 1 | GeneTex (GTX101147) | 1:1000 |
| Rabbit anti ATF4 (D4B8) | Cell Signaling Technology (11815) | 1:1000 |
| Rabbit anti xCT/SLC7A11 (D2M7A) | Cell Signaling Technology (12691S) | 1:1000 |
| Rabbit anti FTH1 | Cell Signaling Technology (3998S) | 1:1000 |
| Rabbit anti IκBα (44D4) | Cell Signaling Technology (4812) | 1:1000 |
| Rabbit anti p-IκBα (Ser 32) (14D4) | Cell Signaling Technology (2859) | 1:1000 |
| Rabbit anti GPX4 | Boster (MB5231) | 1:1000 |
| Rabbit anti IL-1β | Bioworld (BS6067) | 1:1000 |
| HRP-linked GAPDH | KangChen (KC-5G5) | 1:5000 |
| [Anti-rabbit IgG](https://www.cellsignal.cn/products/secondary-antibodies/anti-rabbit-igg-hrp-linked-antibody/7074?site-search-type=Products&N=4294956287&Ntt=hrp-linked+antibody&fromPage=plp), HRP-linked | Cell Signaling Technology (7074S) | 1:2000 |
| [Anti-mouse IgG](https://www.cellsignal.cn/products/secondary-antibodies/anti-rabbit-igg-hrp-linked-antibody/7074?site-search-type=Products&N=4294956287&Ntt=hrp-linked+antibody&fromPage=plp), HRP-linked | Cell Signaling Technology (7076S) | 1:2000 |
| Rabbit anti NF-κB p65 (D14E12) | Cell Signaling Technology (8242T) | 1:200 |
| Alexa Fluor® 488 Donkey Anti-Rabbit IgG (H+L) | Invitrogen (A21206) | 1:2000 |

**Supplementary Table 3: list of RT-qPCR primers.**

| Gene | Forward primer | Reverse primer |
| --- | --- | --- |
| IL-1β | ATGATGGCTTATTACAGTGGCAA | GTCGGAGATTCGTAGCTGGA |
| IL-6 | ACTCACCTCTTCAGAACGAATTG | CCATCTTTGGAAGGTTCAGGTTG |
| TNFα | CTCTTCTGCCTGCTGCACTTTG | ATGGGCTACAGGCTTGTCACTC |
| ATF4 | CCCTTCACCTTCTTACAACCTC | TGCCCAGCTCTAAACTAAAGGA |
| xCT | TCTCCAAAGGAGGTTACCTGC | AGACTCCCCTCAGTAAAGTGAC |
| HO-1 | AAGACTGCGTTCCTGCTCAAC | AAAGCCCTACAGCAACTGTCG |
